# Luminescent materials with dual-mode excitation and tunable emission color for anti-counterfeiting applications

**DOI:** 10.1038/s41598-023-37608-w

**Published:** 2023-07-04

**Authors:** Nina Jaroch, Justyna Czajka, Agata Szczeszak

**Affiliations:** 1 Faculty of Chemistry, Adam Mickiewicz University, Poznań, Uniwersytetu Poznańskiego 8, 61-614 Poznań, Poland; 2 Faculty of Chemical Technology and Engineering, Bydgoszcz University of Science and Technology, Seminaryjna 3, 85-326 Bydgoszcz, Poland

**Keywords:** Chemistry, Materials science

## Abstract

GdVO_4_-based dual-mode phosphors were successfully synthesized via a hydrothermal approach. The X-ray diffraction analysis determined the tetragonal structure as well as *I*4_1_*/amd* space group of products by comparing with a reference pattern no. ICDD #01-072-0277. The morphology of yielded phosphors was confirmed by transmission electron microscopy and scanning electron microscopy. Detailed spectroscopy analysis revealed tunable luminescence properties with an increasing Yb^3+^ content in series of GdVO_4_: x% Yb^3+^, y% Tm^3+^, 5% Eu^3+^ (x = 5, 10, 15, 20; y = 0.1, 0.5, 1) phosphors. For Yb^3+^, Tm^3+^, and Eu^3+^- codoped phosphors we observed bands related to the ^1^G_4_ → ^3^H_6_ and ^1^G_4_ → ^3^F_4_ transitions of Tm^3+^ ions, occurred through the cooperative up-conversion mechanism, where two nearby Yb^3+^ ions were involved in near-infrared absorption. Moreover, the GdVO_4_: 20% Yb^3+^, 0.5% Tm^3+^, 5% Eu^3+^ showed the most outstanding color tunability from red color (x = 0.6338, y = 0.3172) under UV to blue color (x = 0.2640, y = 0.1988) under NIR excitation, which can be applied in anti-counterfeiting activity.

## Introduction

Inorganic materials doped with lanthanide ions (Ln^3+^) play a significant role in many fields of everyday life, based on their numerous applications as lasers, thin film phosphors, in drug delivery, bioimaging, or anti-counterfeiting^1–7^. The last-mentioned group consists of several media in which anti-counterfeiting tags are implemented, such as bar codes, inks, holograms, RFID (Radio Frequency Identification). Every aforementioned security approach has its limitations and thus cannot be applied in i.e., clothes or document markings, which may be regularly treated with water, washing agents, or UV radiation. Recently developed cellulose fibers modified- with inorganic phosphors^8–12^ are prepared via an environmentally friendly NMMO (N-Methylmorpholine-N-Oxide) method. As an outcome, the so-called Tencel fibers may be used for paper modification or as a part of the fabric. Also, during this rigorous process of fibers preparation, the luminescent modifier used has to outstand with its excellent stability. In our research, we chose GdVO_4_-based dual-mode phosphors based on their strong energy absorption as well as the high efficiency of energy transfer processes^13–15^. Another advantage of vanadate-based phosphors as an alternative type of material versus fluoride-based phosphors is their high thermal stability, beneficial in i.e. light-emitting diode application, in which operating temperature exceeds 100 °C^16^. In comparison to the vanadate materials, commonly used in phosphor applications fluoride materials are not only sensitive to high temperatures but also to surface contamination which may accidentally influence spectroscopic properties such as luminescence lifetime or emission color^17^.

Contrary to the accidental influence, the intentional impact on the intensity and different luminescence color under UV or NIR excitation is caused by the specified Ln^3+^ dopant concentration. Rare earth-doped materials exhibiting dual-mode luminescence possess vast potential for various applications^18–21^. When a combination of ions capable of absorbing energy with diverse energy values based on their electron structure is used, a unique luminescence color tunability can be achieved. The perfect combination for obtaining tunable luminescence is the system comprising Yb^3+^, Tm^3+^, and Eu^3+^ ions. Here, the NIR excitation of 980 nm can be absorbed by Yb^3+^ and after transferring two or more photons towards Tm^3+^ and Eu^3+^ ions, a visible up-conversion emission is observed, which is highly dependent on Tm^3+^ and Eu^3+^ ratio. Also, under UV irradiation, there is a possibility of direct Eu^3+^ excitation or energy transfer from the orthovanadate matrix to dopant ions—in both cases, a red emission associated with Eu^3+^ is observed^22,23^.

In this study, we aim to provide a comprehensive understanding of the properties and applications of the luminescent marks based on doped orthovanadates. All of above-mentioned features of the GdVO_4_: Yb^3+^, Tm^3+^, Eu^3+^ phosphors synthesized by the feasible hydrothermal approach, designate as perfect for the anti-counterfeiting applications as the color tunability within the same material is difficult to falsify. The study introduces dual-mode orthovanadates as a viable substitute for fluoride-based materials in the realm of anti-counterfeiting tags. Through precise selection of dopant ions and host matrix, the resulting luminophores undergo up-conversion processes and demonstrate robust emission capabilities owing to charge transfer phenomena between O^2-^ and Eu^3+^ ions. These distinct mechanisms give rise to diverse color emissions and various wavelength excitation. Moreover, the orthovanadate materials exhibit remarkable resilience to harsh environmental conditions, including elevated temperatures, thereby rendering them advantageous by comparison to the aforementioned fluorides, which are more prone to decomposition.

A potential application of the implementation of a orthovanadates modifier into cellulose fibers for paper and fabric markings has been confirmed by the patent application submitted^43,44^. By showcasing the real-world application of our material, we establish its potential for practical implementation and highlight its relevance to various industries. Finally, according to our knowledge, this is the first time that Yb^3+^, Tm^3+^, Eu^3+^ dopants are incorporated into the orthovanadate matrix, and its structural followed by spectroscopic properties are determined. It fills a gap in the existing literature by presenting an innovative approach that has practical implications and opens new avenues for future research and development.

## Experimental section

### Materials

Ammonium metavanadate (NH_4_VO_3_, Sigma Aldrich, 99.9%), gadolinium(III) oxide (Gd_2_O_3_, Standford Materials, 99.99%), ytterbium(III) oxide (Yb_2_O_3_, Standford Materials, 99.99%), thulium(III) oxide (Tm_2_O_3_, Standford Materials, 99.99%), europium(III) oxide (Standford Materials, 99.99%) and acetic acid (CH_3_COOH, POCH, 99,95%) used in the synthesis of the materials.

### Methods

A series of GdVO_4_: x% Yb^3+^, y% Tm^3+^, 5% Eu^3+^ (x = 5, 10, 15, 20; y = 0.1, 0.5, 1) was obtained in hydrothermal conditions. The concentration and the type of dopants was altered based on our knowledge and the literature in order to observe efficient emission processes^24–28^. In addition, the concentration of Yb^3+^, Tm^3+^ and Eu^3+^ dopant ions in prominent GdVO_4_ host was altered to provide the intense, dual-mode luminescence under UV and NIR irradiation for anticounterfeiting applications. The composition of Ln^3+^ ions used was selected to ensure the emission color dependent on excitation wavelength thus considered material is more difficult to replicate.

The synthesis was performed in Berghof autoclave (max. pressure 200 bar, additional stirring). All of the substrates were used as water solutions. The stoichiometric combination of 0.25 M Ln(CH_3_COO)_3_ was mixed with the 0.1 M NH_4_VO_3_ added dropwise under continuous stirring for 30 min. Resultant transparent mixture (pH 4.7) was then transferred to Teflon vessel and put for hydrothermal process under 180 °C for 3 h which yielded with yellow powder. Next, when the autoclave was naturally cooled to room temperature, the product was collected by centrifugation, washed with 1:1 mixture of deionized water and ethanol. Finally, the product was dried at 80ºC for 24 h for further analysis.

### Characterization

The structural analysis was conducted with Bruker AXS D8 Advance powder X-ray diffractometer equipped with Johansson's monochromator and Lynx Eye strip detector, whereas the measurements were performed with Cu-K_α_1 λ = 15,418 Å radiation within the 10–60 2Θ range, 0.05°/s step size. Morphology of studied materials was investigated with the use of transmission electron microscopy, TEM (JEOL 1400 with acceleration voltage of 80 kV) as well as scanning electron microscopy, SEM (Quanta 250 FEG, FEI equipped with EDAX detector). Luminescence properties were studied in terms of photoluminescence and upconversion luminescence, i.e. under UV or NIR excitation. The former was studied with the use of Hitachi F-7000 spectrofluorimeter, equipped with xenon lamp excitation source. The latter phenomenon was studied in terms of emission, luminescence decay and the number of photons involved in the process, with the use of PIXIS:256E Digital CCD Camera equipped with SP-2156 Imaging Spectrograph (Princeton Instruments), Mixed Domain Oscilloscope—200 MHz—Tektronix MDO3022 as well as the excitation source of CNI NIR 2W LASER 975 nm. All of the spectroscopic measurements were conducted at 293 K.

## Results and discussion

### Structure and morphology

**Figure 1:**
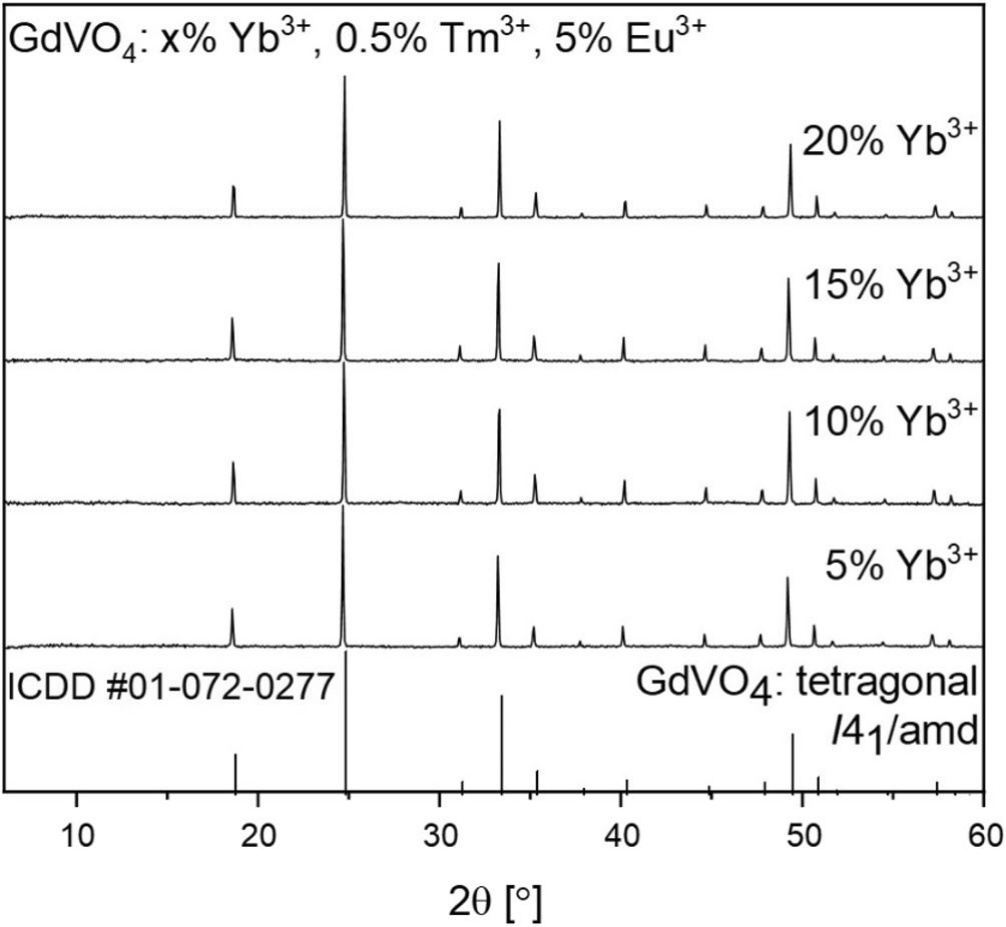
A set of diffraction data for GdVO_4_: x% Yb^3+^, 0.5% Tm^3+^, 5% Eu^3+^ materials.

**Figure 2:**
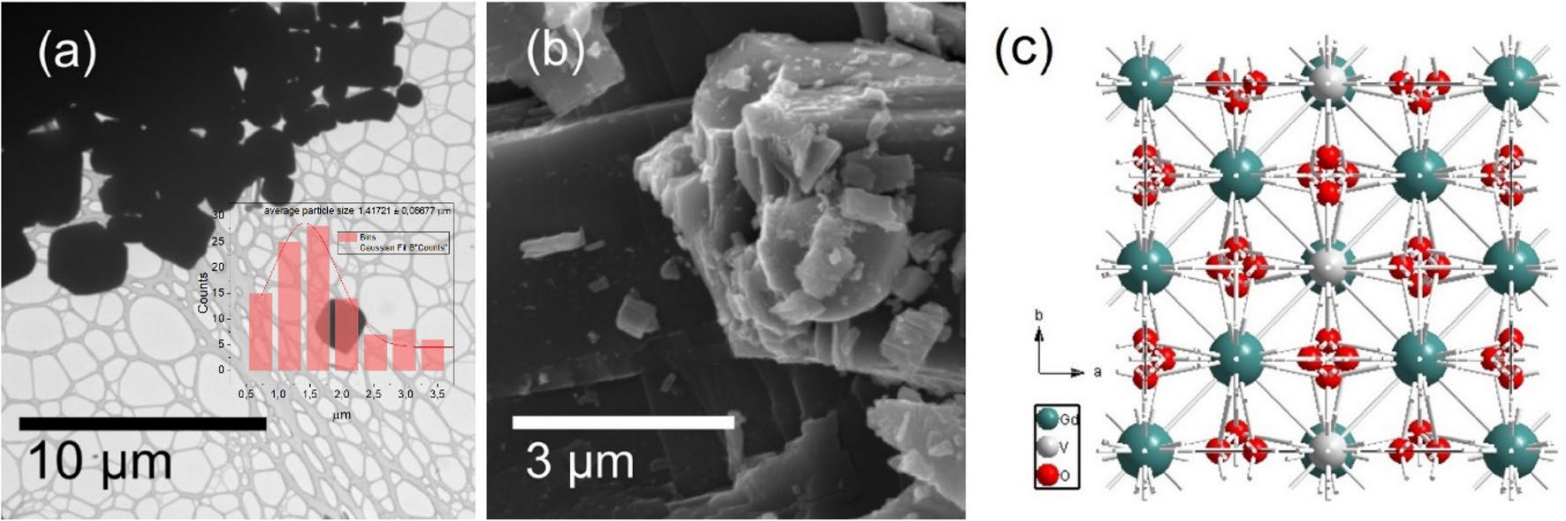
Morphology representation of GdVO_4_: 20% Yb^3+^, 0.5% Tm^3+^, 5% Eu^3+^ sample in terms of TEM with histogram presenting size distribution (**a**) and SEM (**b**) methods and crystal structure of GdVO_4_ I4_1_/amd tetragonal zircontype (ZrSiO_4_) (**c**).

Behind every specific luminescence feature, there are structural and morphological reasons as well. As seen in Fig. 1, the replacement of 25.5% of Gd^3+^ ions with dopant ions in the host structure did not cause severe lattice distortions since the ionic radius of Eu^3+^ is similar, while the Yb^3+^ and Tm^3+^ radii are smaller than the one of Gd^3+^^29^. The synthesized compounds are confirmed to be of GdVO_4_ *I*4_1_/amd tetragonal zircon-type (ZrSiO_4_) structure with the cell parameters of a = b = 7.2126 Å, c = 6.3483 Å, according to the reference pattern no. ICDD #01-072-0277^30,31^. The tetragonal and polyhedral structure of GdVO_4_ are presented in Fig. 2c. Here, vanadium atom of [VO_4_]^3−^ is tetrahedrally coordinated with O^2−^ ions, whereas the Ln^3+^ are surrounded by eight oxygen atoms in a distorted dodecahedron structure^32^. The presence of sharp, narrow reflexes indicates discussed materials as highly crystalline and bulk. By the fact that none of the additional peaks are observed, synthesized powders are monophased and the replacement of Gd^3+^ by Ln^3+^ dopants was successful. What has to be said, an increased grain size growth as well as excellent crystallinity are caused by inevitable high temperature annealing (900 °C) which was applied to induce the UC luminescence, initially diminished by structure defects, typical for materials synthesized in hydrothermal conditions^33^. These features were further confirmed with the use of TEM and SEM methods (Fig. 2a,b). The obtained orthovanadate crystals were observed to be agglomerated, displaying an irregular morphology. The average grain size, determined from the broadest fraction, was found to be approximately 1.5 µm. These observations confirm the microstructural properties of the orthovanadate crystals and provide quantitative data regarding their size distribution illustared on histogram (inset Fig. 2a). Despite the large grain size, it was possible to disperse the powder in water for further use in cellulose fiber modification.

### Spectroscopic properties

**Figure 3:**
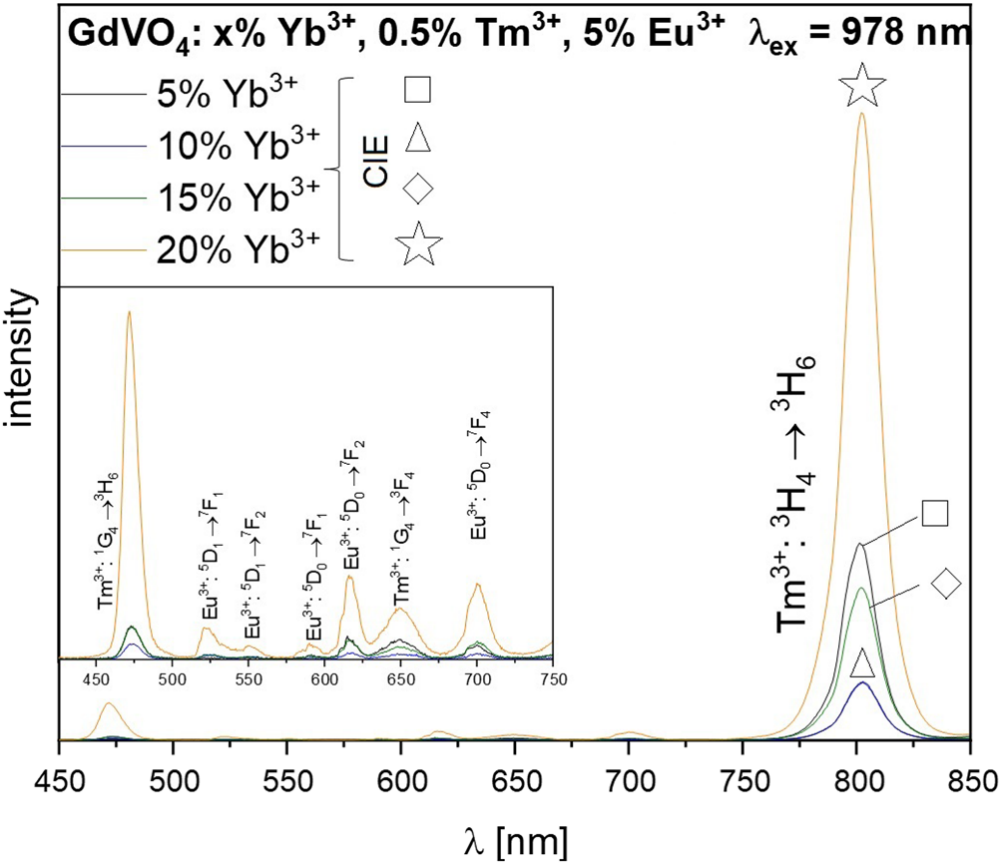
UC luminescence spectra for GdVO_4_: x% Yb^3+^, 0.5% Tm^3+^, 5% Eu^3+^ samples recorded under CW 975 nm excitation.

**Figure 4:**
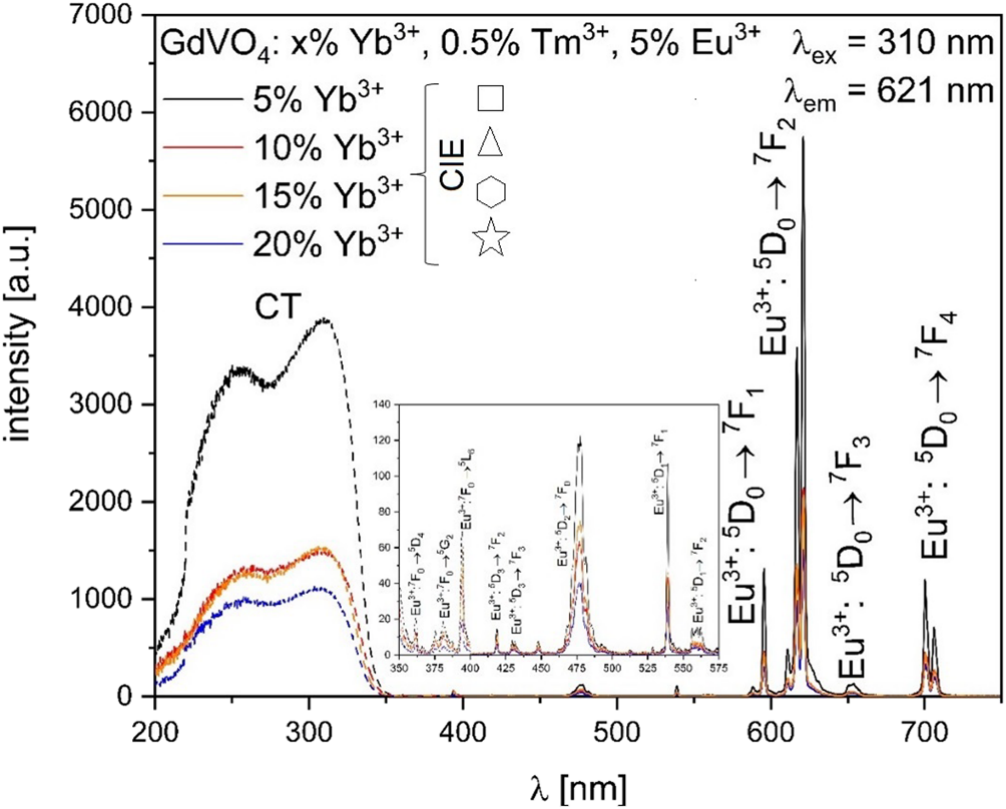
PLE (dashed line) and PL (solid line) spectra of GdVO_4_:x%Yb^3+^, 0.5%Tm^3+^, 5%Eu^3+^ materials.

According to the Figs. 3 and 4, GdVO_4_: 20% Yb^3+^, 0.5% Tm^3+^, 5% Eu^3+^ sample was chosen amongst the series as the most promising sample thus used for the fabric preparation based not only on its diverse luminescence color under UV and NIR excitation, but also the outstanding UC emission in the visible range.

### Photoluminescence

Based on the excitation spectrum in the UV range Fig. 4, the O^2−^–Eu^3+^ charge transfer band (CT) with the maximum at 310 nm was chosen to observe visible luminescence at 621 nm, assigned to the ^5^D_0_ → ^7^F_2_ transition. What has to be mentioned, the broad CT band is in fact combined of O^2−^–V^5+^ and O^2−^–Eu^3+^; however, based on the small difference in between O^2−^ and V^5+^, as well as the large charge difference, O^2−^–V^5+^ in [VO_4_]^3−^ is easier observed^34^. Also, there are additional weak f–f transitions typical for Eu^3+^ ions in the 200–500 nm range^35,36^. Based on the ionic radii difference^34^, Gd^3+^ is being replaced by Eu^3+^ thus in the GdVO_4_: Yb, Eu, Tm system, Eu^3+^ has D_2d_ symmetry as it is surrounded by eight O^2-^ ions. Relative intensity of ^5^D_0_ → ^7^F_1_ and ^5^D_0_ → ^7^F_2_ is altered based on the local site symmetry of the Eu^3+^ ions^11,37,38^. In this research, the intensity of hypersensitive ^5^D_0_ → ^7^F_2_ is the highest among the Eu^3+^ emission bands which indicated the low symmetry around Eu^3+^ ions^38^. What is more, with an increasing Yb^3+^ concentration, the intensity of both excitation and emission curves is decreasing in terms of the Eu^3+^ → Yb^3+^ energy transfer, since the distance between these ions is shortened^38^. This phenomenon is further confirmed by the calculated Eu^3+^ luminescence lifetime values (Table 1) monitored under 310 nm excitation. With the increasing Yb^3+^ concentration, the energy is migrating from Eu^3+^ excited states to Yb^3+^ ions.

**Table 1:** A set of calculated luminescence lifetime values for GdVO_4_: x% Yb^3+^, 0.5% Tm^3+^, 5% Eu^3+^ phosphors under 310 nm excitation.

Luminescence lifetime [μs]					
Sample	Eu^3+^: ^5^D_2_ → ^7^F_0_ (~ 476 nm)	Eu^3+^: ^5^D_0_ → ^7^F_1_ (~ 596 nm)	Eu^3+^: ^5^D_0_ → ^7^F_2_ (~ 621 nm)	Eu^3+^: ^5^D_0_ → ^7^F_3_ (~ 654 nm)	Eu^3+^: ^5^D_0_ → ^7^F_4_ (~ 700 nm)
GdVO_4_: 5% Yb^3+^, 0.5% Tm^3+^, 5% Eu^3+^	171 ± 5	342 ± 5	282 ± 2	347 ± 6	289 ± 1
GdVO_4_: 10% Yb^3+^, 0.5% Tm^3+^, 5% Eu^3+^	176 ± 5	285 ± 2	306 ± 3	308 ± 3	280 ± 2
GdVO_4_: 15% Yb^3+^, 0.5% Tm^3+^, 5% Eu^3+^	176 ± 5	318 ± 5	296 ± 4	320 ± 6	260 ± 2
GdVO_4_: 20% Yb^3+^, 0.5% Tm^3+^, 5% Eu^3+^	175 ± 5	298 ± 5	281 ± 3	325 ± 7	171 ± 5

**Figure 5:**
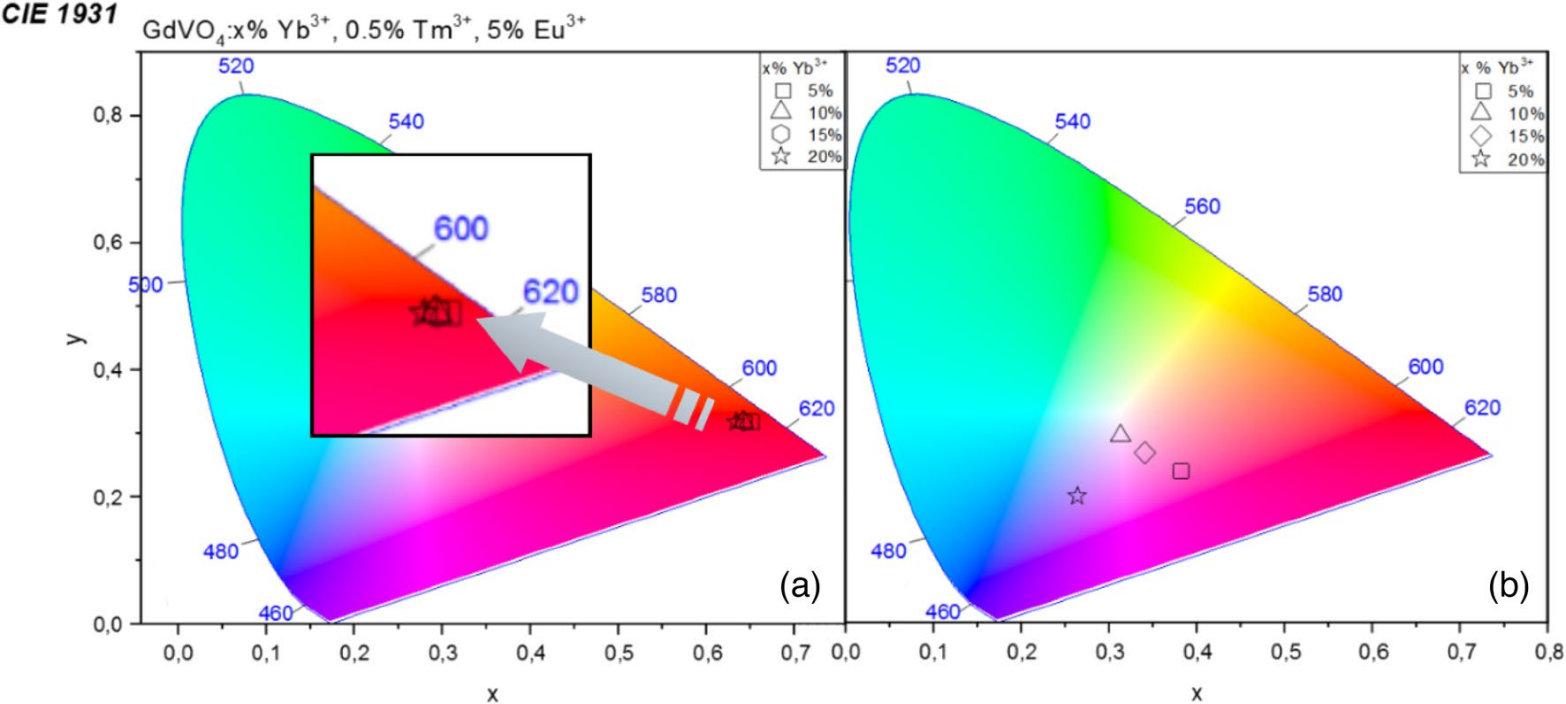
Chromaticity diagram for GdVO_4_: x% Yb^3+^, 0.5% Tm^3+^, 5% Eu^3+^ samples under 310 nm with an enlarged inset (**a**) and CW 975 nm excitation (**b**).

According to the chromaticity diagram Fig. 5, under 310 nm excitation the outcoming luminescence color is not altered by the incorporated Tm^3+^ ions.

### Upconversion luminescence

It is essential for dual-mode luminescence (ergo inimitable anti-counterfeiting materials) to be intense both under UV and NIR irradiation. According to the upconversion spectrum depicted in Fig. 4, the energy transfer between Eu^3+^ and Tm^3+^ ions is observed as variety of Eu^3+^ and Tm^3+^ emission bands are present in the spectrum. What has to be noted, the population of Eu^3+^ via inefficient phononassisted- Yb^3+^–Eu^3+^ is barely observed; here, Tm^3+^ acts as an energy mediator between the sensitizer (Yb^3+^) and emitter (Eu^3+^)^22^. According to that, several emission bands associated to the Tm^3+^ and Eu^3+^ are observed in the spectrum, which vary with intensity, namely ^1^G_4_ → ^3^H_6_ (Tm^3+^, ~ 478 nm), ^5^D_1_ → ^7^F_1_ (Eu^3+^, ~ 521 nm), ^5^D_1_ → ^7^F_2_ (Eu^3+^, ~ 552 nm), ^5^D_0_ → ^7^F_1_ (Eu^3+^, ~ 590 nm), ^5^D_0_ → ^7^F_2_ (Eu^3+^, ~ 615 nm), ^1^G_4_ → ^3^F_4_ (Tm^3+^, ~ 650 nm), ^5^D_0_ → ^7^F_4_ (Eu^3+^, ~ 700 nm) as well as the most intense ^3^H_4_ → ^3^H_6_ (Tm^3+^, ~ 800 nm) band. The last-mentioned transition is observed in NIR region of spectrum thus it does not influence the emission color. What has to be mentioned, throughout changing Yb^3+^ concentration, the chromaticity coordinates of synthesized materials change, according to the Fig. 5 and Table 2. With an increasing Yb^3+^ content, the luminescence color shifts from the red towards purple and blue region of CIE chromaticity diagram. By the gradual substitution of Gd^3+^ by Yb^3+^, the distance between sensitizer and emitters such as Tm^3+^, Eu^3+^ decrease. As mentioned before, the efficiency of Yb^3+^–Eu^3+^ transfer is low thus the Yb^3+^–Tm^3+^ transfer is favored here. In this case, the competitive Tm^3+^–Eu^3+^ absorption is decreased, which is also connected with the lower intensity of Eu^3+^ emission bands in the UC spectrum, as well as the red component of the luminescence color^23^. Also, when Yb^3+^–Tm^3+^ is greatly enhanced, the relative intensity between blue and red emissions of Tm^3+^ is increased, which results with the outcoming blue upconversion luminescence^40,41^.

**Table 2:** A set of calculated chromaticity coordinates (x,y) for GdVO_4_: x% Yb^3+^, 0.5% Tm^3+^, 5% Eu^3+^ phosphors under 310 nm and 975 nm excitation.

Sample	Chromaticity coordinates (x, y)	
	UV	NIR
GdVO_4_: 5% Yb^3+^, 0.5% Tm^3+^, 5% Eu^3+^	0.6495, 0.3202	0.3808, 0.2418
GdVO_4_: 10% Yb^3+^, 0.5% Tm^3+^, 5% Eu^3^	0.6424, 0.3183	0.3134, 0.2942
GdVO_4_: 15% Yb^3+^, 0.5% Tm^3+^, 5% Eu^3+^	0.6431, 0.3181	0.3411, 0.2672
GdVO_4_: 20% Yb^3+^, 0.5% Tm^3+^, 5% Eu^3+^	0.6338, 0.3172	0.2640, 0.1988

**Figure 6:**
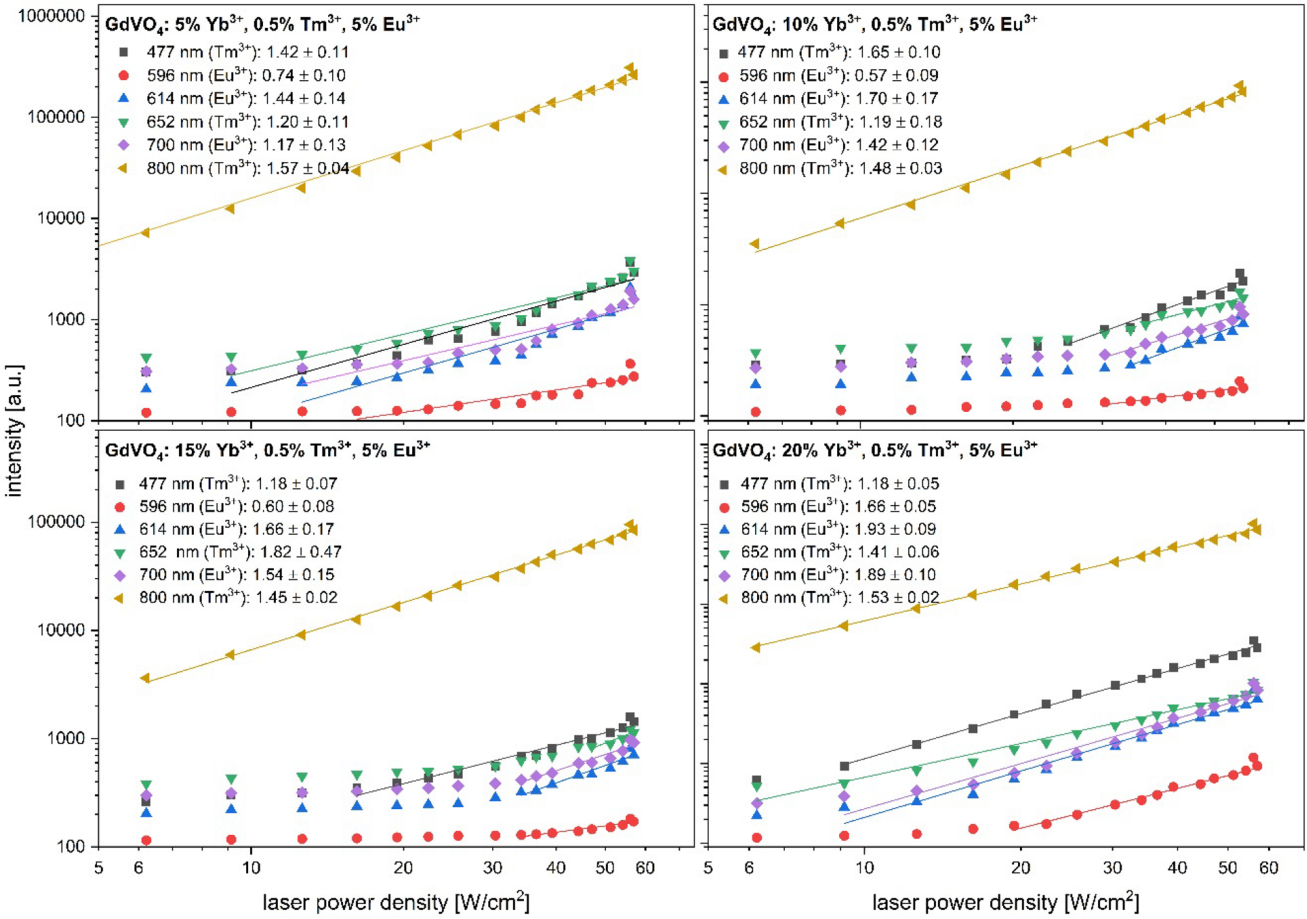
Laser power density studies for GdVO_4_: x% Yb^3+^, 0.5% Tm^3+^, 5% Eu^3+^ samples recorded under CW 975 nm excitation.

The population of Eu^3+^ and Tm^3+^ excited levels, as well as the energy transfer between these species were studied in terms of luminescence decay. Here, due to the color change, the most important bands are the ones in the blue and red region of spectrum. Based on that, the greatest attention was put to the lifetime of ^1^G_4_ → ^3^H_6_ (Tm^3+^, ~ 478 nm), ^5^D_0_ → ^7^F_2_ (Eu^3+^, ~ 615 nm) and ^1^G_4_ → ^3^F_4_ (Tm^3+^, ~ 650 nm). As shown in Table 3, the luminescence lifetime of Eu^3+^ is decreasing with an increasing Yb^3+^ content. Based on that, the possibility of Tm^3+^–Eu^3+^ is diminished, whereas Yb^3+^–Tm^3+^ transfer is favored. It is also confirmed by the enhanced blue emission of Tm^3+^ since less energy is transferred towards Eu^3+^ site of lattice. Moreover, in order to derive the average number of photons (n) involved in the upconversion process, a laser power dependent luminescence study was performed (Fig. 6). Interestingly, the slopes (n) of ^1^G_4_ → ^3^H_6_ and ^1^G_4_ → ^3^F_4_ transitions suggest the involvement of two photons regardless the Yb^3+^ concentration. In the contrary to the common understanding of the ^1^G_4_ population mechanism, i.e. through threephoton- absorption, meant also as sequential sensitization, in the case of GdVO_4_: x% Yb^3+^, 0.5% Tm^3+^, 5% Eu^3+^ a cooperative sensitization is in fact happening. In this mechanism there are two Yb^3+^ ions involved, which absorbs photons in order to promote themselves towards ^2^F_5/2_ excited state. Then, a coupled cluster state of two Yb^3+^ ions formed transfers energy towards Tm^3+^ which results with the population of its ^1^G_4_ level^42^.

**Table 3:** A set of calculated luminescence lifetime values for GdVO4: x% Yb^3+^, 0.5% Tm^3+^, 5% Eu^3+^ phosphors under 975 nm excitation.

Luminescence lifetime [μs]						
Sample	Tm^3+^: ^1^G_4_ → ^3^H_6_ (~ 476 nm)	Eu^3+^: ^5^D_1_ → ^7^F_1_ (~ 521 nm)	Eu^3+^: ^5^D_0_ → ^7^F_2_ (~ 615 nm)	Tm^3+^: ^1^G_4_ → ^3^F_4_ (~ 650 nm)	Eu^3+^: ^5^D_0_ → ^7^F_4_ (~ 700 nm)	Tm^3+^_:_ ^3^H_4_ → ^3^H_6_ (~ 800 nm)
GdVO_4_: 5% Yb^3+^, 0.5% Tm^3+^, 5% Eu^3+^	85 ± 5	88 ± 2	229 ± 5	99 ± 5	442 ± 1	88 ± 3
GdVO_4_: 10% Yb^3+^, 0.5% Tm^3+^, 5% Eu^3+^	89 ± 2	81 ± 5	225 ± 3	87 ± 3	204 ± 2	89 ± 2
GdVO_4_: 15% Yb^3+^, 0.5% Tm^3+^, 5% Eu^3+^	92 ± 2	98 ± 2	207 ± 4	97 ± 6	93 ± 2	88 ± 2
GdVO_4_: 20% Yb^3+^, 0.5% Tm^3+^, 5% Eu^3+^	95 ± 1	85 ± 2	180 ± 3	95 ± 1	158 ± 1	91 ± 1

**Figure 7:**
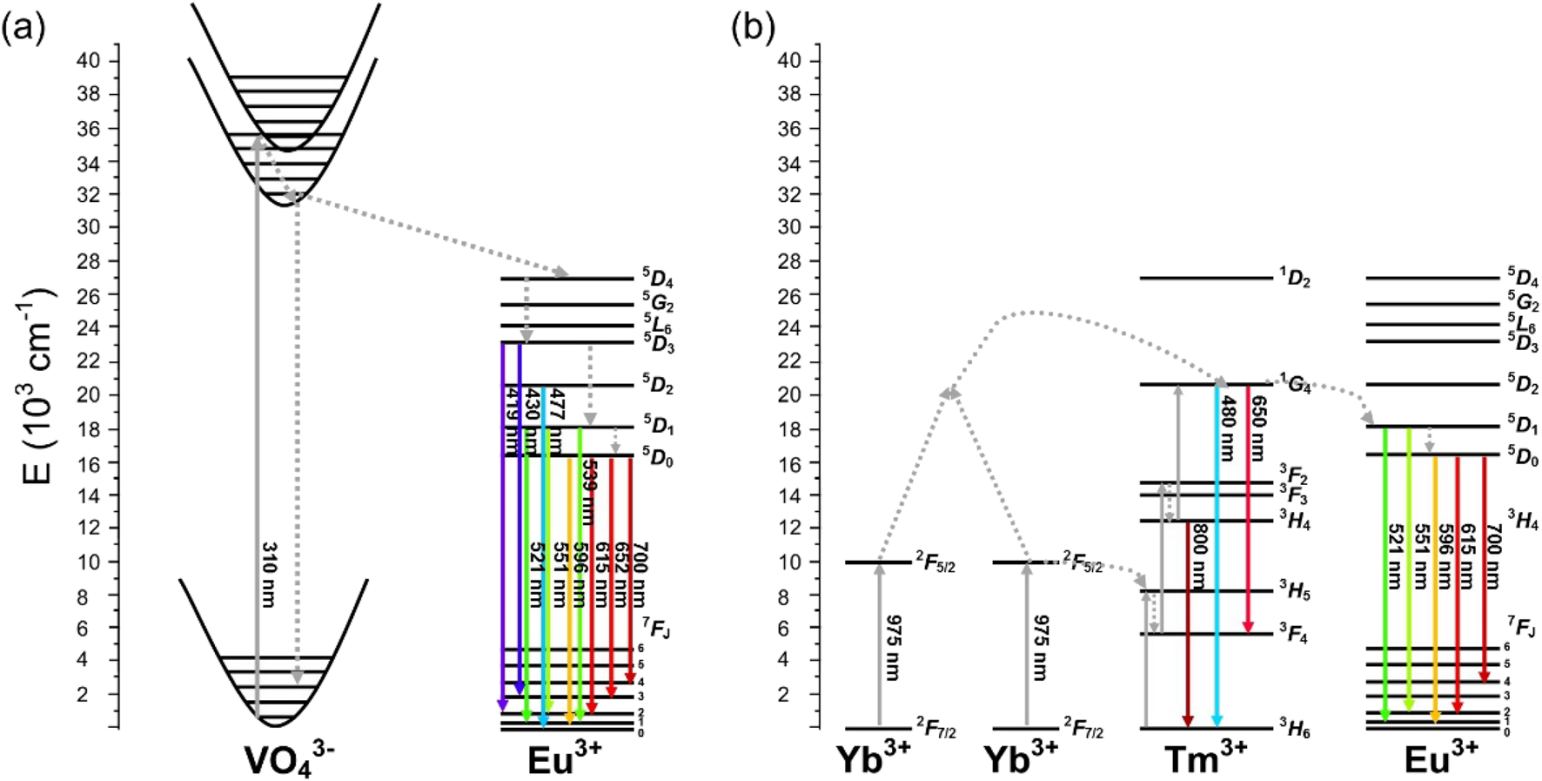
Energy level diagrams of Yb^3+^, Tm^3+^ and Eu^3+^ ions and possible energy transfer (**a**) and UC (**b**) mechanism.

To summarize all studied processes, a mechanism of upconversion in GdVO_4_: x% Yb^3+^, 0.5% Tm^3+^, 5% Eu^3+^ systems can be proposed in Fig. 7. The whole phenomenon begins under 975 nm CW excitation, when the energy is first absorbed by two nearby Yb^3+^ ions. This results with the promotion of sensitizers from their ^2^F_7/2_–^2^F_5/2_ level. At this point, two simultaneous processes are happening. Due to the formation of coupled Yb^3+^ cluster state, another photon is absorbed and transferred towards ^1^G_4_, from which 478 nm and 650 nm emissions occur. Also, there is a photon transferred from this level towards Eu^3+ 5^D_1_, where after energy dissipation to ^5^D_0_, several emissions associated with Eu^3+^ are observed in the spectrum. What is more, the Tm^3+ 3^H_5_ is populated via photon transfer from Yb^3+ 2^F_5/2_. After nonradiative relaxation to ^3^H_4_, an 800 nm emission is observed.

**Figure 8:**
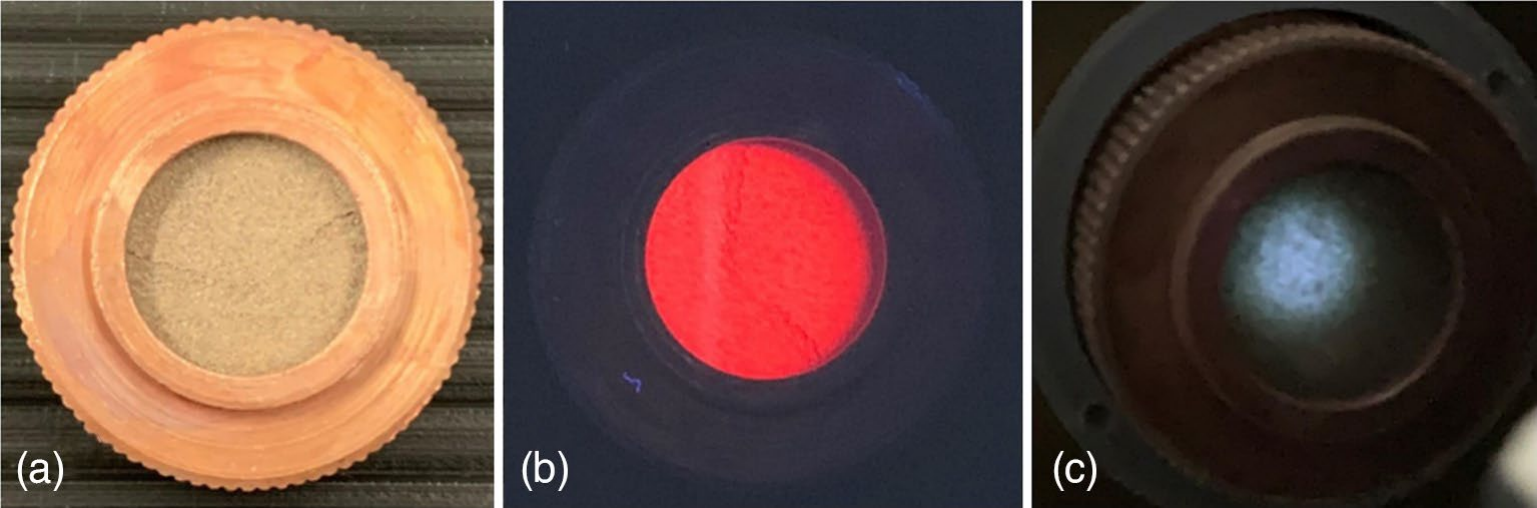
Actual images of GdVO_4_: 20% Yb^3+^, 0.5% Tm^3+^, 5% Eu^3+^ powder in daylight (**a**), under 254 nm UV excitation (**b**) and 975 nm IR excitation (**c**).

Based on its pristine tunable luminescence properties, sample composed of GdVO_4_: 20% Yb^3+^, 0.5% Tm^3+^, 5% Eu^3+^ was chosen for fibers preparation. Then luminescent fibers were used for paper modification and fabric production as an example of anti-counterfeiting application^43,44^. Regardless the medium, the outcoming luminescence color as well as its intensity remain unchanged. These properties recommend GdVO_4_: 20% Yb^3+^, 0.5% Tm^3+^, 5% Eu^3+^ phosphor for anticounterfeiting applications performed in patent proposal submission. In Fig. 8, an actual luminescence color under different excitation sources is present.

## Conclusions

To conclude, the pre-eminent, spectroscopic properties of GdVO_4_: 20% Yb^3+^, 0.5% Tm^3+^, 5% Eu^3+^ define this material as the excellent one for anti-counterfeiting purposes. In our study, we investigated different Yb^3+^ concentration and its influence on structural and spectroscopic properties. With an increased sensitizer content, the upconversion luminescence is more intense whereas its color is tuned towards blue region of spectrum. What is more, in Yb^3+^/Tm^3+^/Eu^3+^ system, ^1^G_4_ → ^3^H_6_ and ^1^G_4_ → ^3^F_4_ emissions result from twophoton excitation in terms of cooperative sensitization where two nearby Yb^3+^ ions are involved in IR excitation absorption process. Also, varied luminescence color under different sources of excitation qualified GdVO_4_: 20% Yb^3+^, 0.5% Tm^3+^, 5% Eu^3+^ phosphor for anticounterfeiting application. In applied cellulose medium, luminescence color and intensity remained unchanged in comparison to the phosphor in the powder state.

## Data Availability

The datasets generated during and analyzed during the current study are available from the corresponding author on reasonable request.
